# Hematemesis and gastric pseudo-obstruction secondary to an iatrogenic diaphragmatic hernia

**DOI:** 10.1055/a-2480-3710

**Published:** 2024-12-10

**Authors:** Kui Xu, Fan Wang, Yan-Min Yang, Xiao Yuan, Chao-Chao Yang, Xiang-Lin Hao, Cong Yuan

**Affiliations:** 1 485308 Gastroenterology, Peopleʼs Hospital of Yuxi City, Yuxi, China; 2 485308 Medical Imaging, Peopleʼs Hospital of Yuxi City, Yuxi, China; 3 117913 Gastroenterology and Digestive Endoscopy Center, Affiliated Hospital of North Sichuan Medical College, Nanchong, China

A 29-year-old woman was referred with recurrent nonbilious vomiting for 2 weeks and fresh hematemesis for the past 12 hours. Her past medical history included left nephrectomy, splenectomy, and partial diaphragmatic resection and repair 3 years previously owing to left renal tuberculosis with extensive adhesions.

Emergency esophagogastroduodenoscopy revealed significant fresh blood in the lumen and multiple mucosal erosions in the lower esophagus and greater curvature of the stomach, along with gastric distortion and distal pseudo-obstruction ( Fig. 1 **a, b** ; Video 1 ). Repeated gastroscopic attempts to pass through the obstruction revealed a normal appearance of the gastric antrum without blood staining ( Fig. 1 **c** ). A nasogastric tube was placed. A contrast study showed a left-sided diaphragmatic hernia and distal gastric obstruction ( Fig. 2 ). Computed tomography scans confirmed the left-sided diaphragmatic defect with herniation of the dilated gastric fundus and body, and part of the colon and mesentery into the left thorax, accompanied by collapse of the gastric antrum beneath the diaphragm ( Fig. 3 ). An incarcerated diaphragmatic hernia was suggested, and an emergency exploratory laparotomy was performed. Intraoperatively, the gastric serosa appeared normal in color, with no signs of strangulation. The herniated organs were reduced, and the diaphragmatic defect was repaired. The patient’s recovery was uneventful postoperatively.

**Fig. 1:**
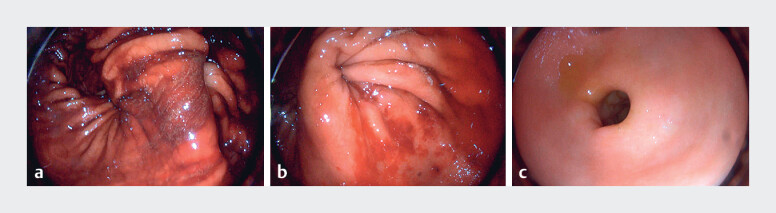
Endoscopic images of the stomach during emergency esophagogastroduodenoscopy showing: **a** fresh blood in the lumen and distortion of the stomach; **b** pseudo-obstruction in the lower gastric body; **c** a normal appearance of the gastric antrum with no blood staining.

**Fig. 2:**
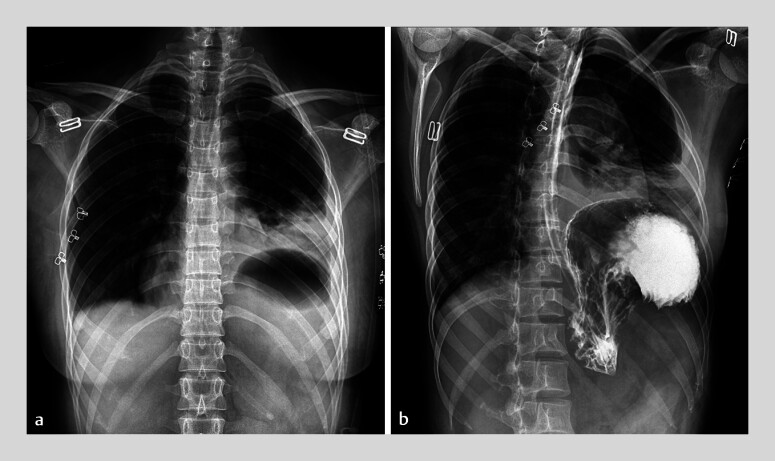
Upper gastrointestinal series with administration of oral iohexol contrast showing a left-sided diaphragmatic hernia, with herniation of the proximal stomach and failure of contrast medium to enter the distal stomach.

**Fig. 3:**
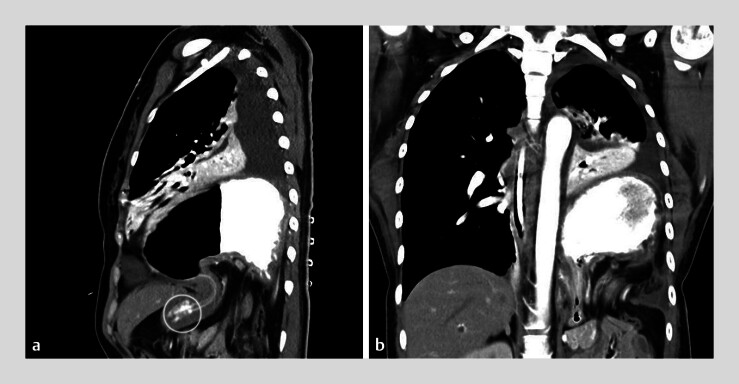
Computed tomography images showing a diaphragmatic hernia containing the dilated gastric fundus and body in the left hemithorax, compression of the left lung, and a pleural effusion, accompanied by collapse of the gastric antrum (circle) beneath the diaphragm on: **a** sagittal view; **b** coronal view.

Emergency esophagogastroduodenoscopy revealed significant fresh blood, multiple mucosal erosions in the lower esophagus and stomach, distortion of the lumen and distal pseudo-obstruction, with a contrast study and computed tomography scans confirming the presence of a diaphragmatic hernia.Video 1

A diaphragmatic hernia involves the protrusion of abdominal contents into the thorax through a defect in the diaphragm, which may be congenital or acquired 1 . Acquired hernias are prevalent among adults, often resulting from trauma, whether iatrogenic or non-iatrogenic. In this patient, prior surgery may have weakened her diaphragm, which led to the diaphragmatic hernia 2 . The gastric fundus and body herniated into the thorax and were incarcerated at the diaphragmatic defect, causing distal gastric pseudo-obstruction. Prolonged vomiting caused multiple erosions in the esophageal and gastric mucosa, resulting in hematemesis. In summary, prompt recognition and surgical intervention for an incarcerated hernia can help yield a favorable prognosis 1 .

Endoscopy_UCTN_Code_CCL_1AB_2AC_3AC

## Notes

Endoscopy E-Videos https://eref.thieme.de/e-videos
*E-Videos* is an open access online section of the journal *Endoscopy* , reporting on interesting cases and new techniques in gastroenterological endoscopy. All papers include a high-quality video and are published with a Creative Commons CC-BY license. Endoscopy E-Videos qualify for HINARI discounts and waivers and eligibility is automatically checked during the submission process. We grant 100% waivers to articles whose corresponding authors are based in Group A countries and 50% waivers to those who are based in Group B countries as classified by Research4Life (see: https://www.research4life.org/access/eligibility/ ). This section has its own submission website at https://mc.manuscriptcentral.com/e-videos .
